# Effects of day-time exposure to different light intensities on light-induced melatonin suppression at night

**DOI:** 10.1186/s40101-015-0067-1

**Published:** 2015-07-04

**Authors:** Tomoaki Kozaki, Ayaka Kubokawa, Ryunosuke Taketomi, Keisuke Hatae

**Affiliations:** Faulty of Design, Kyushu University, 4-9-1 Shiobaru, Fukuoka city, Minami-ku Japan; Graduate School of Design, Kyushu University, 4-9-1 Shiobaru, Fukuoka city, Minami-ku Japan

**Keywords:** Light, Light intensity, Melatonin, Day-time, Night-time

## Abstract

**Background:**

Bright nocturnal light has been known to suppress melatonin secretion. However, bright light exposure during the day-time might reduce light-induced melatonin suppression (LIMS) at night. The effective proportion of day-time light to night-time light is unclear; however, only a few studies on accurately controlling both day- and night-time conditions have been conducted. This study aims to evaluate the effect of different day-time light intensities on LIMS.

**Methods:**

Twelve male subjects between the ages of 19 and 23 years (mean ± S.D., 20.8 ± 1.1) gave informed consent to participate in this study. They were exposed to various light conditions (<10, 100, 300, 900 and 2700 lx) between the hours of 09:00 and 12:00 (day-time light conditions). They were then exposed to bright light (300 lx) again between 01:00 and 02:30 (night-time light exposure). They provided saliva samples before (00:55) and after night-time light exposure (02:30).

**Results:**

A one-tailed paired *t* test yielded significant decrements of melatonin concentration after night-time light exposure under day-time dim, 100- and 300-lx light conditions. No significant differences exist in melatonin concentration between pre- and post-night-time light exposure under day-time 900- and 2700-lx light conditions.

**Conclusions:**

Present findings suggest the amount of light exposure needed to prevent LIMS caused by ordinary nocturnal light in individuals who have a general life rhythm (sleep/wake schedule). These findings may be useful in implementing artificial light environments for humans in, for example, hospitals and underground shopping malls.

## Introduction

Light is thought to have the most powerful impact on the human circadian rhythm and sleep/wake cycle. Prior to artificial lighting, humans adjusted their circadian rhythm to match the natural 24 h bright/dark cycle (i.e. the earth’s rotation). In modern society, however, bright environments still exist at night while, thanks to modern buildings and underground spaces, dim light environments are possible during the day. Modern humans, especially in urban areas, are hardly ever exposed to a 24-h bright/dark cycle. The modern light environments possibly cause the desynchronization of our circadian rhythm and 24-h life rhythm.

Bright nocturnal light has been known to suppress melatonin secretion [1–4], and the International Agency for Research on Cancer (IARC) has classified work shifts that involve circadian disruption as a probable carcinogenic factor (Group 2A). Because melatonin may have potent anti-cancer activity [5, 6], light-induced melatonin suppression (LIMS) at night may increase the risk of cancer. Some research has found that LIMS occurs at night even under low light intensities (120–200 lx) [7, 8], which would suggest even greater health risks.

Yet bright light at day-time has been thought to increase melatonin secretion [9–11] and may reduce LIMS at night. Hebert et al. [12] evaluated LIMS after day-time exposure to different light conditions for 1 week. The subjects were exposed to bright light (both by being outside and by using a light box inside) and wore dark goggles (that allowed about 2 % light transmission) during the day-time. Significantly, more LIMS was found after subjects experienced dim light conditions than when they experienced bright conditions. Smith et al. [13] measured LIMS after different day-time light stimuli (200 vs. 0.5 lx) for 3 days. LIMS significantly increased after dim light exposure compared with bright light exposure. Yet another study measured LIMS in indoor and outdoor workers and reported a significant correlation between the percentage of LIMS and the subjects’ average light exposure in a 24-h period [14].

These findings suggest that a bright/dark cycle is necessary for health. To maintain an adequate secretion of melatonin, it is important to understand the effective proportion of day- to night-time light. However, this proportion is unknown because there have been very few studies on accurately controlling both day- and night-time light conditions. This study, therefore, evaluates the effect of different day-time light intensities on LIMS. In this study, the light intensity of night-time light is set at 300 lx, based on ordinary light conditions in a home.

## Materials and methods

### Subjects

Twelve male subjects aged 19–23 years (mean ± S.D., 20.8 ± 1.1) gave informed consent to participate in this study. All subjects were free of medical conditions at the time of the experiment, and none had a history of psychiatric or sleep disorders. The subjects were instructed to abstain from alcohol and caffeine beginning 1 day before the experiment began and to maintain a regular sleep/wake schedule (24:00 to 08:00) beginning 7 days before the experiment. The sleep wake schedule was confirmed by a wrist-worn actigraph (Actiwatch-L 9 (AWL); Mini-Mitter, Bend, OR, USA).

### Experimental procedure

**Fig. 1:**
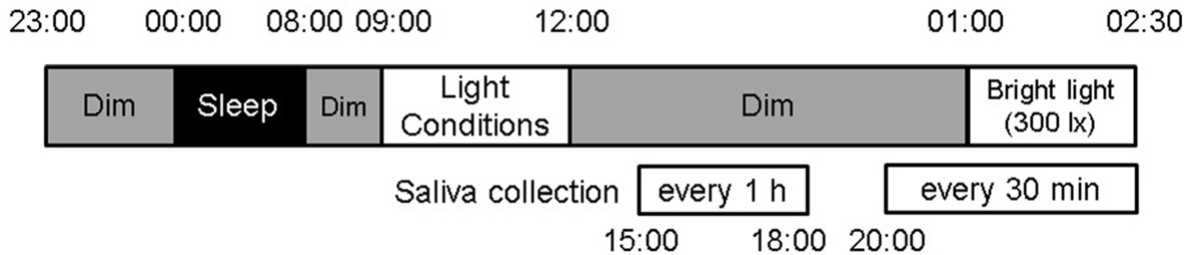
Time schedule. The subjects were exposed to different light conditions (<10, 100, 300, 900 and 2700 lx) from 09:00 to 12:00 and to bright light (300 lx) from 01:00 to 02:30

The experiments were carried out from August 2012 to November 2012; the experimental schedule is shown in Fig. 1. The subjects arrived at the experimental chamber before 23:00 and were exposed to dim light conditions (<10 lx). They slept in the experimental chamber under dark conditions until 08:00 and then had breakfast until 09:00 under the same dim conditions experienced prior to sleep. After breakfast, the subjects experienced light conditions from 09:00 to 12:00. After this exposure, the subjects had lunch until 13:00 under dim light conditions, which were maintained until 01:00, and provided saliva samples every hour from 15:00 to 18:00 in order to determine the threshold of dim light melatonin onset (DLMO) [15, 16]. The subjects were allowed to watch movies and read books in the experimental chamber; however, the illuminance of the movie displays was kept at less than 2 lx, because bright light from movie displays may suppress melatonin secretion [17]. Then, the subjects had dinner and showered from 18:00 to 20:00. Saliva samples were collected under dim light conditions every 30 min from 20:00 to 01:00, and the subjects experienced bright light (300 lx) exposure for 90 min from 01:00 to 02:30. After exposure to bright light, the subjects provided saliva samples. They were instructed to maintain a sitting position on their chairs during saliva collection because posture might affect melatonin secretion [18].

For each subject, exposure to the five different light conditions (<10, 100, 300, 900 and 2700 lx) were conducted in five different experimental sets with one light condition per set, namely, repeated measure design. Experiments were performed under different conditions in random order at intervals of more than 5 days. Although the subjects conducted their normal life during the intervals, they were instructed to maintain a regular sleep/wake schedule (24:00 to 08:00). The ambient temperature in the experimental chamber was kept at 27 °C.

### Light conditions

**Fig. 2:**
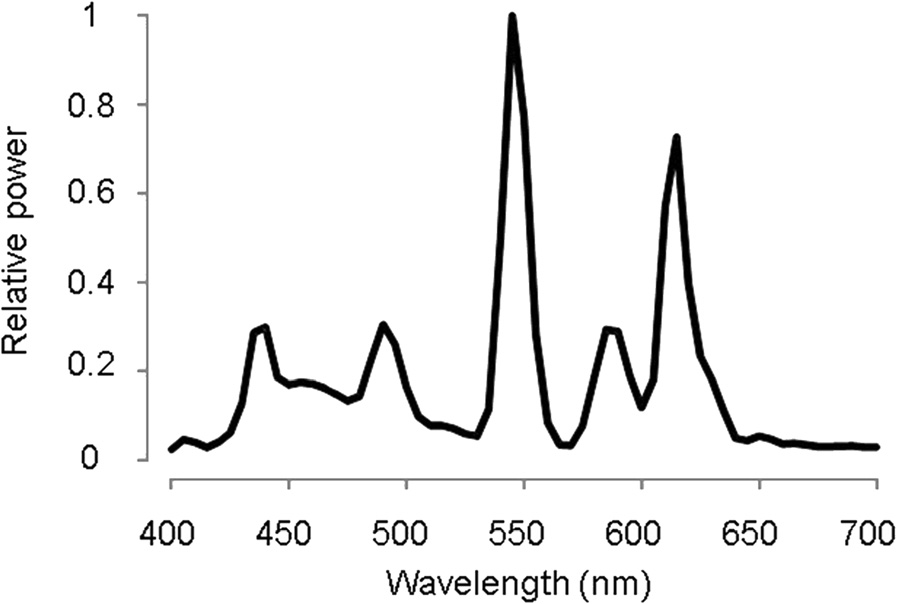
The spectral distribution of the light source

Intensities of <10, 100, 300, 900 and 2700 lx were used for the experiment’s day-time light conditions (‘Light conditions’ in Fig. 1). Day- and night-time light conditions (‘Bright light’ in Fig. 1) were achieved by using a white fluorescent lamp (Panasonic Ltd. Co., Osaka, Japan), which was placed in front of each subject. The correlated colour temperature, general colour rendering index and chromaticity coordinates (*x*, *y*) of the light source were 4523 K, 86 and (0.362, 0.381), respectively, as measured by a spectroradiometer (Lightspec, GretagMacbeth, NY, USA). Figure 2 shows the relative spectral power distributions. The vertical illuminance level of each light condition at the cornea was set using an illuminance metre (HD 2012.2, Delta Ohm, Padua, Italy).

### Salivary melatonin assay

Saliva samples were collected using non-cotton (polypropylene–polyethylene complex swab) collection devices (Sarstedt K.K. Numbrecht, Germany), because cotton swabs might interfere with the assay results of salivary melatonin [19]. Saliva samples were centrifuged at 1500 *g* for 5 min and then frozen at −30 °C until assayed. Melatonin levels in the samples were analysed using a commercially available Radioimmunoassay (RIA) kit (Direct Saliva Melatonin RIA; Buhlmann Laboratories, Allschwil, Switzerland). The kit’s limit of detection was 0.9 pg/mL, and its limit of quantification was 0.2 pg/mL. The mean intra- and inter-assay coefficients of variance were 7.9 and 9.8 %, respectively.

### Data analysis

Since there is substantial inter-individual variation in endogenous melatonin levels [20, 21], a salivary threshold was determined by calculating the mean of the four base melatonin values plus twice the standard deviation of these values [15]. In this study, four base melatonin values were obtained from the saliva samples collected from 15:00 to 18:00 for each light condition. The thresholds varied among subjects and ranged from 0.68 to 3.2 pg/mL (mean ± S.D., 1.6 ± 0.9). The time of the DLMO phase was determined by linear interpolation between the time points before and after melatonin concentration increased and remained above the thresholds [15, 16]. Data on two subjects were excluded from the analysis because DLMO times were not detected during the sampling period (20:00–01:00); two subjects showed lower melatonin levels than the thresholds during the sampling period. Thus, results for only 10 subjects were analysed.

The DLMO phases were compared for each light condition using a one-way repeated-measured analysis of variance (ANOVA). To assess the differences in melatonin concentration over time and between pre- and post-nocturnal light exposure, data were analysed by using a two-way, within-subject factor, repeated-measured ANOVA. For post hoc analyses, comparing the melatonin concentration between pre- and post-bright light exposure under each light condition, a one-tailed paired *t* test was used. These statistical analyses were performed using SPSS version 16.0 (SPSS, Chicago, IL, USA). Differences in which *p* was <0.05 were considered statistically significant. Power analysis were also performed to calculate the type II error probability (β) for each statistical analyses using G*Power version 3.1 [22], because the sample size of this study (*n* = 10) was relatively small.

## Results

**Fig. 3:**
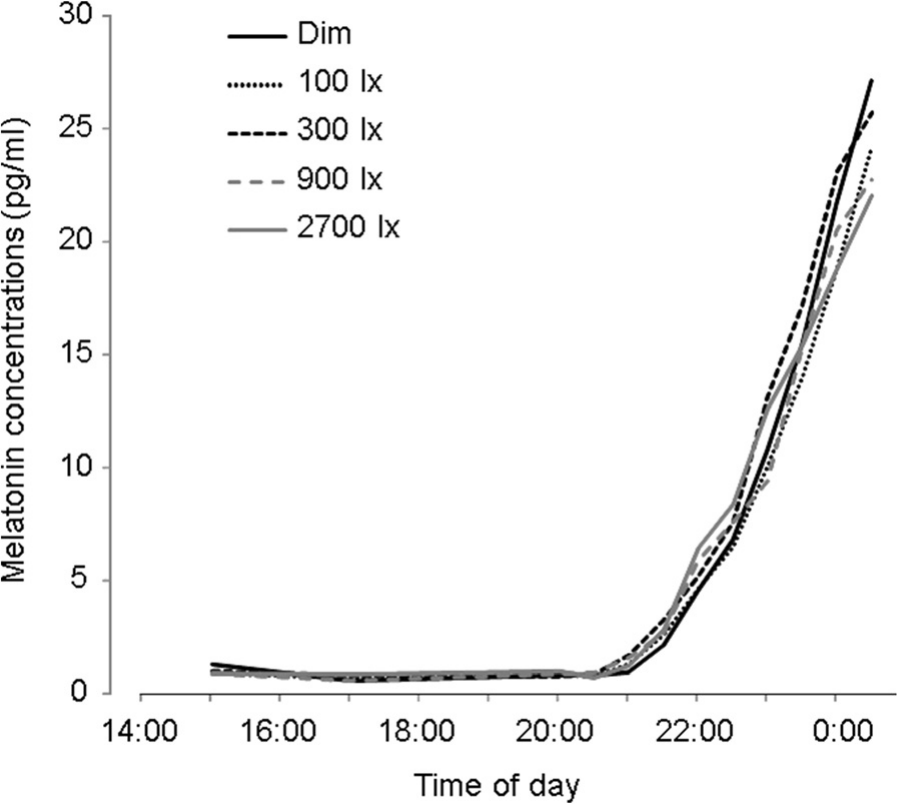
The time courses for mean melatonin concentrations under the five different light conditions from 15:00 to 0:30

**Fig. 4:**
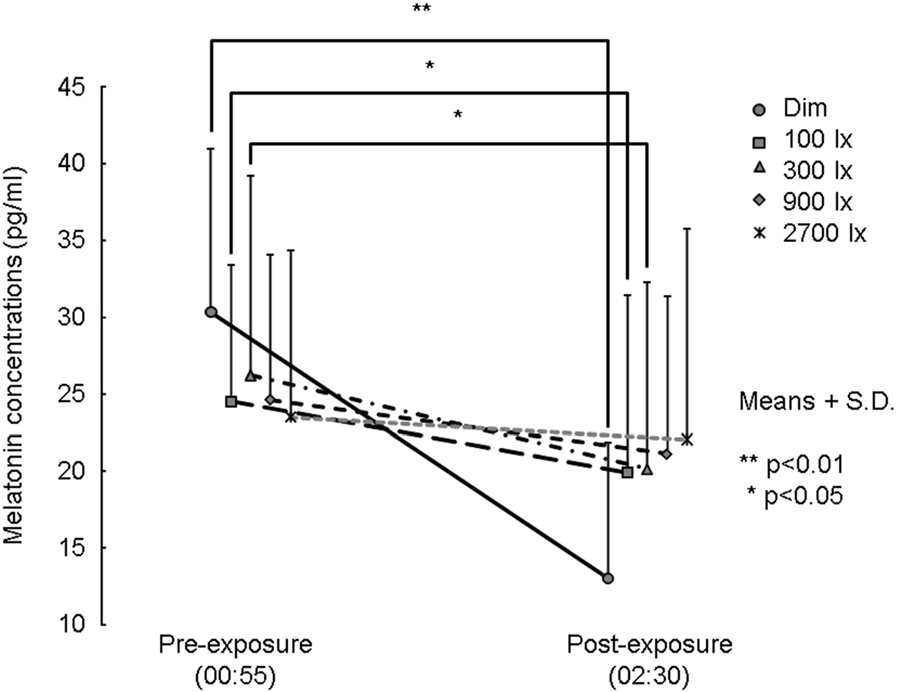
Mean (+S.D.) melatonin concentrations of each morning light condition before (pre-exposure) and after (post-exposure) the nocturnal light exposure

The time courses for mean melatonin concentrations under the five different light conditions are shown in Fig. 3. There were no significant differences in the DLMO phase for each light condition (*F*_1.36_ = 1.4, n.s., *β* = 0; Table 1). Figure 4 shows mean melatonin concentrations of each morning light condition before (pre-exposure) and after (post-exposure) the nocturnal light exposure. Two-way, repeated-measured ANOVA for the experiment period demonstrated that time intervals have a significant effect on melatonin secretion (*F*_1.9_ = 15.8, *p* < 0.01, *β* = 0) and interact with light conditions (*F*_4.36_ = 5.4, *p* < 0.01, *β* = 0), and light conditions do not have a significant effect (*F*_4.36_ = 0.10, n.s., *β* = 0.37). Post hoc comparison revealed significant melatonin decrements after night-time light exposures under day-time dim, 100 and 300 lx light conditions (dim; *t*_9_ = 8.7, *p* < 0.01, *β* = 0: 100 lx; *t*_9_ = 1.9, *p* < 0.05, *β* = 0.47: 300 lx; *t*_9_ = 1.9, *p* < 0.05, *β* = 0.46). No significant differences were present in the melatonin concentrations between night-time pre- and post-light exposure of day-time 900 and 2700 lx light conditions (900 lx; *t*_9_ = 1.1, n.s., *β* = 0.73; 2700 lx; *t*_9_ = 0.4, n.s., *β* = 0.90).

**Table 1:** Mean DLMO times (time of day) for each light condition. Standard deviations (S.D.) are in minutes

Light conditions	DLMO
Dim	22:15 (69)
100 lx	22:10 (70)
300 lx	21:58 (72)
900 lx	21:52 (76)
2700 lx	21:51 (57)
	Mean (S.D.)

## Discussion

There were no significant differences in melatonin concentrations between night-time pre- and post-light exposure under day-time 900 and 2700 lx light conditions; however, melatonin concentrations significantly decreased after night-time light exposure under day-time dim light conditions. The subjects were instructed to maintain a regular sleep/wake schedule (beginning sleep at 00:00 and waking at 08:00) 7 days before the experiment began. Mean DLMO times showed no significant differences among day-time light conditions and occurred at about 22:00. The subjects were thought to have a circadian rhythm synchronized with a general life rhythm because DLMO occurred about 2 h before sleep began at 00:00 [23]. Present findings suggest the amount of light exposure needed to prevent LIMS caused by ordinary night-time light in individuals who have a general life rhythm (sleep/wake schedule).

Melatonin concentration before night-time light exposure was high under day-time dim light condition; however, no significant differences were present on the melatonin concentrations among day-time light conditions. The high melatonin concentration may be caused by tryptophan in the breakfast. Melatonin concentration was increased by having tryptophan-rich breakfast with exposure to bright light at day-time [24]. In this study, melatonin levels were compared pre- and post-three-day experiment; the subjects had tryptophan-rich breakfast and exposure to bright light at day-time for 3 days. Therefore, ingested tryptophan before present experiment possibly had an impact on melatonin secretion.

One possible reason that diurnal light exposure limits LIMS is that, as has been suggested in previous research [25, 26], non-visual photoreceptors (melanopsin-containing retinal ganglion cells (mRGCs)) are mainly responsible for mediating LIMS. In an animal study [27], light-induced desensitization of mRGSs was measured after 52 min of dark- and bright light conditions. The response amplitude to 5-s light pulse was reduced after the bright background light. Another animal study [28] showed a significant increase in melanopsin expression under prolonged exposure of constant darkness. Prolonged exposure of constant bright light, on the other hand, decreased melanopsin expression. Although whether these animal findings can accurately explain these conditions remains to be seen, the response of mRGCs and/or melanopsin expression may be one of the reasons that LIMS is prevented by diurnal light exposure.

Because melatonin concentrations showed no significant decrease after night-time light exposures of bright light conditions during the morning (>900 lx), we may conclude that day-time light exposure may be needed more than night-time light exposure to prevent LIMS. A one-tailed paired *t* test was used to decrease Type II error probabilities because of small sample size. The probabilities, however, were high under day-time 900 and 2700 lx light conditions (900 lx; *β* = 0.73; 2700 lx; *β* = 0.90). These high probabilities imply that no significant decrease of melatonin concentration is obtained after night-time light exposure in other subjects. Although no significant differences were present among the mean DLMO obtained at each separate light condition, variations were present among the individual DLMOs obtained for separate subjects within each light condition. Phase dependence of the light-induced phase shift has been reported as a phase response curve (PRC) [29–31]. According to the PRC, the phase advancing effect of the light is greater at 2–6 h after the circadian phase at 0 h (CP0); it is lesser within 2 h after CP0. Given that the preventing effect of diurnal light on LIMS also has a PRC, lower doses of diurnal light exposure may prevent LIMS in individuals who have a later sleep schedule.

## Conclusions

Although our findings are limited, the present study provides application data that suggest that day-time exposure to a minimum dose of light can prevent LIMS even within just 1 day. These findings may be useful as humans adapt to artificial light environments in, for example, hospitals and underground shopping malls. Light-induced nocturnal melatonin suppression, however, depends on the duration of light exposure as well as the light intensity [32]. The wavelength composition of nocturnal light also affects light-induced melatonin suppression [8, 33–35]. In addition, photosensitivity may be involved based on the basis of the person’s eye colour [36] and ageing [37]. Therefore, further research is needed to examine the parameters of light exposure and the photosensitive variation of human beings.
